# The dynamics of the endothelial glycocalyx: a rare snapshot by electron microscopy

**DOI:** 10.1007/s10456-023-09880-x

**Published:** 2023-05-15

**Authors:** Carolin C. Drost, Andreas Unger, Wolfgang A. Linke, Hans Vink, Philipp Kümpers

**Affiliations:** 1 Department of Medicine D, Division of General Internal Medicine, Nephrology, and Rheumatology, University Hospital Münster, Münster, Germany; 2 Institute of Physiology II, University Hospital Münster, Münster, Germany; 3 Department of Physiology, Cardiovascular Research Institute Maastricht, Maastricht University, Maastricht, The Netherlands; 4 Microvascular Health Solutions, Alpine, USA

## Introduction

**Fig. 1:**
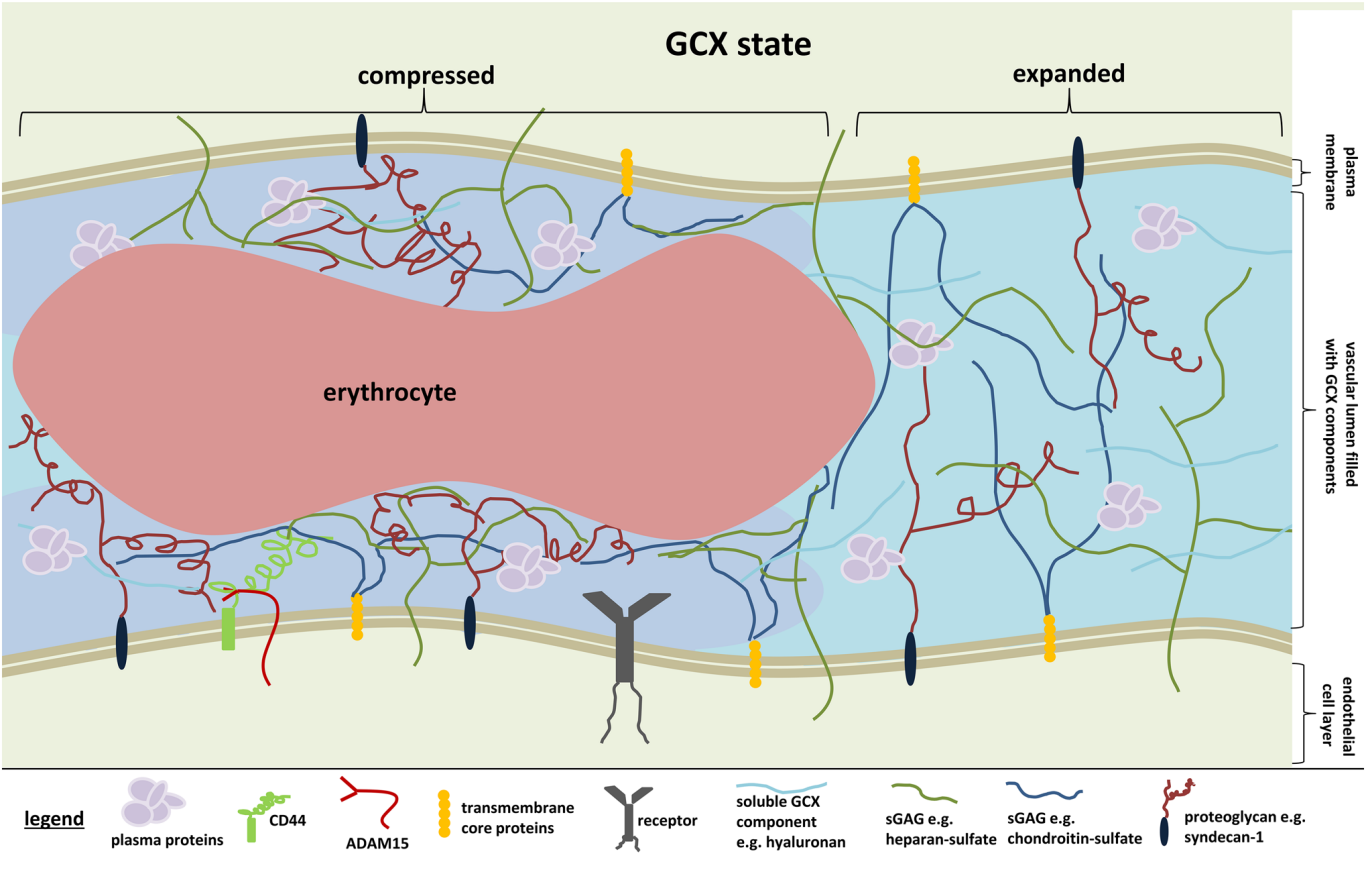
Simplified schematic of capillary GCX. The GCX is a mesh of carbohydrate-rich structures such as (sulfated) glycosaminoglycans (sGAG), proteoglycans and soluble components of variable composition that covers the entire vascular tree. Due to constant shear stress and the passage of blood cells, highly dynamic properties with variable conformational changes, compression and (re-)expansion are characteristic features attributed to this layer. In addition to its mechanistic and barrier properties, the GCX contributes functionally to the regulation of vascular homeostasis, the uptake of plasma proteins and the transduction of signals

In recent years, the endothelial glycocalyx (GCX) has emerged as a structure of increasing interest in diseases with predominant vascular dysfunction [1, 2]. Consisting mainly of glycosaminoglycans (GAG), proteoglycans (PG) and soluble components, it covers the entire endothelium and represents the first part of the vascular barrier (Fig. 1). The regulation of vascular homeostasis, the uptake of plasma proteins and the transduction of signals in response to shear stress are just some of the many physiological functions now attributed to this delicate layer [3]. The consequences of disrupted GCX are striking in diseases of systemic inflammation. In studies of sepsis and trauma patients, elevated plasma levels of GCX components such as syndecan-1, hyaluronan or heparan-sulfate (HS) indicate GCX damage early in the disease process. The normally well-balanced quiescent vascular state is transformed into a pro-inflammatory phenotype with a predominance of signaling molecules promoting endothelial dysfunction – as exemplified by the shift from vascular protective Tie2-activating ligand Angpt-1 to its deactivating antagonist Angpt-2 [4]. The resultant reduced GCX coverage facilitates adhesion of leucocytes [5] and weakens the vascular barrier function, leading to progressive plasma extravasation, which is exacerbated by a subsequent cellular response of junctions loosening [4, 6]. The full development of vascular leakage marks the onset of edema formation, hypotension and ultimately organ failure. Sufficient therapeutic approaches to break this vicious cycle are lacking, but are urgently needed as the lethality of these entities remained high in recent years [7]. Notably, the emerging Sars-CoV-2 pandemic in 2019 joins the group of high impact diseases with predominant vascular dysfunction [8]. Therefore, a strong involvement of GCX in COVID-19 is plausible, as especially in severe forms, the course of disease is strikingly parallel to bacterial sepsis with end-stage development of acute respiratory distress syndrome [9]. Numerous studies show increased markers of GCX damage in COVID-19 and innovative new in vivo methods for estimating GCX constitution via sublingual video microscopy provide evidence that GCX alterations in COVID-19 are comparable to those observed in bacterial sepsis [10–12]. Recent studies also suggest that the prominent coagulopathy-associated complications of COVID-19, such as thrombosis, may well be a result of GCX-related impaired fibrinolysis [13]. In terms of experimental approaches, protection or rescue of GCX might be a valuable strategy to sufficiently counteract the progression of systemic inflammation [14–16]. Therefore, it is essential to understand and unravel the interplay of this highly variable and heterogenous layer. In particular, reliable visualization of GCX remains a challenge and is subject of the current study.

## Past and present approaches to GCX visualization

Although studies from the early 1940s already suggested the existence of such a structural layer between endothelial cells and the vascular lumen [17], it was not until the late 1960s and the development of electron microscopy (EM) that its existence was visualized and confirmed [18]. The development of innovative methods such as intravital microscopy now offers the possibility of studying this highly dynamic structure even in intact and vital organisms. Years of research have painted a picture of the GCX as it is commonly described today: a gel-like layer of fragile components, yet robust as a whole despite the constant influence of blood flow. Reliable experiments form the basis of this concept. However, it should be noted that the majority of in vivo data are based on sophisticated methods in which the thickness of the GCX is estimated rather than measured [19].

Although individual GCX components have been deciphered down to their molecular sugar sequences, it is unlikely that the constantly changing shape of the GCX has been faithfully visualized.

Immunofluorescence (IF) staining allows selective inference of the composition of specific GCX components under varying conditions and can be used to estimate GCX coverage on endothelial cell surfaces. However, it lacks the resolution to provide insight into the ultrastructural arrangement of the GCX and therefore provides a very precise but rather qualitative view of GCX composition [20, 21].

Atomic force microscopy provides the resolution to quantitatively measure the thickness of the GCX on living cells or tissues [22, 23], but visualization remains difficult, although new approaches appear promising [24].

To get an idea of the “true” GCX dimensions, in vivo applicable methods such as the FITC-dextran exclusion zone technique [5] or the non-invasive sublingual side-stream dark field microscopy [25] are state of the art as they exclude artefacts of sample processing. Even if only based on indirect GCX estimation, real-time assessment of GCX dimensions under physiologic conditions with blood-pressure dependent blood flow and varying plasma compositions in living organisms is of immense value as such conditions are difficult to reproduce in ex vivo settings. The high susceptibility of in vitro GCX to external conditions needs to be considered and openly discussed when such data are interpreted [26]. However, to date there is no method available to directly visualize GCX in living organisms.

EM is the only method that allows adequate insight into the ultrastructure of the GCX, but given the variety of appearances in literature – from hairy bundles to unstructured conglomerates – a high susceptibility to artefacts during perfusion, fixation and sample processing seems likely, complicating interpretation [27, 28]. In particular, the high risk of GCX dehydration during processing for EM is a known confounding factor that limits the reliability of GCX thickness and coverage measurements [29]. Nevertheless, the unique feature of high-resolution GCX visualization combined with quantitative assessment of GCX composition highlights EM as an outstanding and essential method in GCX research.

## Methods and results

In the current study, EM imaging was performed on slices of peripheral kidney tissue from healthy C57BL/6 J wild-type mice. All experiments were approved by the local authorities (Landesamt für Natur, Umwelt und Verbraucherschutz NRW, Germany). Ketamine/xylazine anesthesia was applied before perfusion with 5 ml freshly prepared fixative solution (pH 7.3, sodium cacodylate 0.1 M, 2% glutaraldehyde, 2% lanthanum, 2% sucrose) via the left ventricle for 2 min. The left kidney was explanted immediately after perfusion, stored in fixative overnight at 4 °C and transferred to a glutaraldehyde-free solution the next day for further preparation. Samples were treated with osmium tetroxide, counterstained with uranyl acetate in 70% ethanol, dehydrated, and embedded in Durcupan resin. Resin blocks were prepared, and ultrathin sections were cut with a Leica Ultracut S (Mannheim, Germany). Sections were adsorbed onto glow-discharged Formvar carbon-coated copper grids. Images were taken using a Zeiss LEO 910 electron microscope (Zeiss, Oberkochen, Germany) equipped with a TRS sharpeye CCD camera and manufacturer’s software (Troendle, Moorenweis, Germany).

**Fig. 2:**
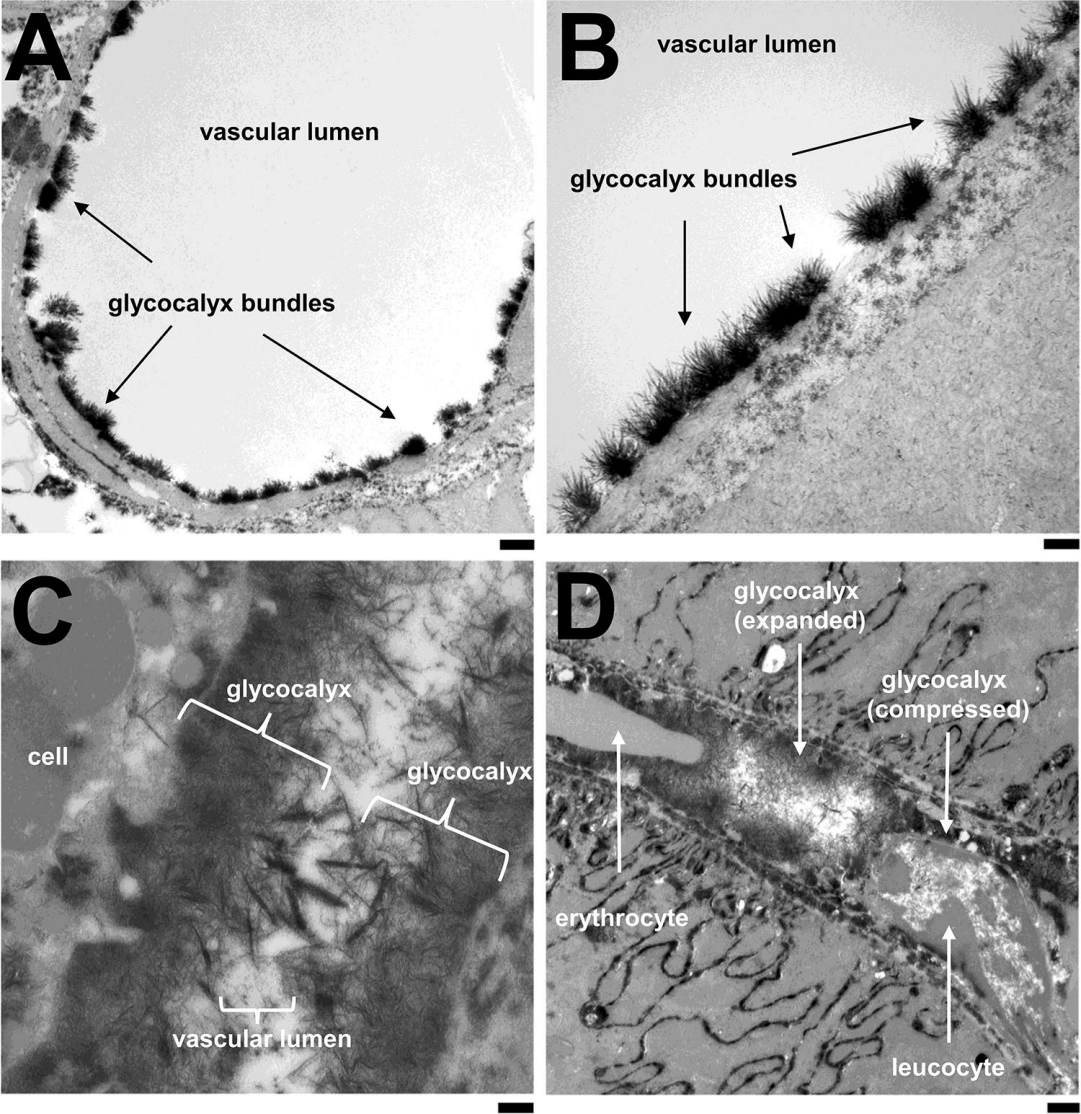
Electron microscopy images of GCX in murine kidney vessels. EM imaging shows GCX bundles in larger vessels of murine kidneys with an average thickness of about 250 nm (**A** + **B**). In capillary vessels a dense GCX-compatible layer continuously covers the endothelial surface and fills nearly the whole lumen, reaching a thickness of up to 800 nm. Passing blood cells cause compression of GCX which appears (re-)expanded in-between again (**C** + **D**). Scale bar in A and D is 500 nm and 100 nm B and C

This approach to visualize the GCX in murine renal vessels (Fig. 2) produced images that, to our knowledge, are the first to be convincingly consistent with the concept of GCX described above. In larger vessels, we first observed dense, but undoubtedly hairy and at the same time delicate glycocalyx bundles with an average thickness of about 250 nm (Fig. 2A, B). Such bundles have been described in numerous publications, but it is still not clear whether they represent the entire GCX or only the lower core of this layer bound to the plasma membrane. When we looked at peritubular capillaries, we found an even more compact glycocalyx-compatible layer that continuously covered the endothelium and filled almost the entire lumen. The surprisingly high thickness of about 800 nm exceeded the height of the individual tufts in the large vessels by a factor of 3 (Fig. 2C, D).

Some of the indirect methods for measuring GCX take advantage of the more or less pronounced compression of the glycocalyx by circulating blood cells and estimate the thickness of the GCX from the resulting variability in the lateral deflection of these cells [10]. However, the dynamics in the *ultra*structure of the GCX cannot yet be visualized methodically. Fortunately, we were able to capture exactly this (compressive) passage of circulating blood cells through the dense capillary GCX in a rare EM snapshot (Fig. 2D). Coincidentally, a passing nucleus-carrying blood cell (e.g. leukocyte) and an erythrocyte can be seen in the capillary lumen. However, the GCX between the two passing blood cells is not compressed at all. Assuming that one of the two cells must have already passed through the capillary, this indicates a very rapid and complete re-expansion of the GCX.

## Conclusion

Within the last decades the GCX has emerged as a relevant structure of interest, particularly in the context of vascular pathophysiology. Increasing research interest has led to the adaptation of conventional methods as well as the development of new innovative methodological approaches that have sharpened the image of the GCX as it is described today. To fully unravel the complex and highly dynamic properties of this elusive layer, a combination of the existing methods is inevitable. The constant effort to optimize and develop new approaches ideally leads to consolidation and confirmation of the concepts developed. Given the fragility of the GCX and the methodological limitations discussed above, a little bit of luck may also be helpful. For now, we can conclude that this rare EM snapshot convincingly supports the current concept of blood cells squeezing through a gel-like capillary glycocalyx, which has emerged from dynamic but indirect studies of the GCX.
